# A Lorentz variant theory that passes fundamental tests of special relativity and makes diverging, testable but as of yet untested predictions

**DOI:** 10.12688/f1000research.129133.1

**Published:** 2023-04-17

**Authors:** Daniël Bischoff van Heemskerck

**Affiliations:** 1 Institute Lorentz of Theoretical Physics, Leiden University, Leiden, The Netherlands

**Keywords:** Special Relativity, Fundamental physics, Superluminal jets of matter

## Abstract

**Background**: Tests of special relativity have been conducted over the past century with increasing accuracy and none have showed violations of Lorentz invariance. In this paper we will examine whether these tests are together sufficient to rule out theories that violate observational symmetry.

**Methods:** A variant theory is outlined where relativistic effects such as length contraction and time dilation are purely local consequences of the relative velocity between a system and its medium. The outlined theory is tested against the fundamental tests of special relativity.

**Results:** It is found that although this alteration does not align with the principle of relativity, it quantitatively aligns with the experimental results of the fundamental tests of special relativity and their modern variations, and makes diverging, testable but as of yet untested predictions concerning Doppler shift and time dilation.

**Conclusions:** These results warrant a closer theoretical inspection of the outlined theory, and could provide a direction to test for new physics. A modified Ives-Stilwell experiment is proposed to test between this model and special relativity.

## Introduction

Although all our physical theories are ultimately based on assumptions, some of our theories have been so predictive and well-tested that it’s easy to mistake their underlying assumptions for proven laws of nature. Perhaps the foremost example of this is the principle of relativity. Of course the principle is fundamental to both the special ^1^ and the general theory of relativity developed by Einstein in the early 20th century, as well as to Lorentz’ ether theory, ^2^^,^^3^ but even before that it had been an integral part of physics for almost three hundred years, being a cornerstone of Newtonian mechanics. The principle was formulated first by Galileo in 1632. In his ‘Dialogue Concerning the Two Chief World Systems’ ^4^ he argues one cannot measure absolute motion. Using a thought experiment concerning a ship that sails in uniform motion on a perfectly smooth sea, he showed that an observer below deck would observe all to be the same as when the ship had not been moving at all.

Examining Galileo’s thought experiment we can say it is no coincidence that it took place within the ship as opposed to on deck. If the ship had been in motion relative to the medium of air it was sailing through, physical experiments conducted on deck would be affected by that relative motion. Of course one could still not ascertain if the ship was absolutely in motion or the air was, but there would be no observational symmetry between the reference frame at rest relative to the air and one in motion. Observational symmetry between two reference frames in relative motion seems to necessitate at least one of two conditions to be true: Either the medium surrounding the reference frames needs to be locally co-moving with the reference frames, or the reference frames need to be within a vacuum.

In this paper we will assume that there is no such thing as vacuum, but that every single part of is space if ‘filled’ with matter, so that every body may be considered to be surrounded by a material medium. If all space contains matter, all motion through space naturally influences observation. As such, the principle of relativity would not be a general law at all, only a special case that arises when special local conditions are met.

In essence the inquiry underlying this paper is simple. Could the relativistic effects we have directly or indirectly measured be caused not by the relative motion between a body and an observer, but by the relative motion between a body and its material medium?

## The basic assumptions

Let us assume there to be no empty space in our universe and also, *ad hoc*, that as the local velocity between a reference frame and its medium increases, quantities such as time, length and energy in that frame will differ in magnitude from the same quantities observed in a frame at rest relative to its medium by a factor of:

$$\gamma =\frac{1}{\sqrt{1-\frac{v^{2}}{c^{2}}}}$$

Where v is the velocity of a reference frame relative to its medium and c is the (unattainable) velocity of a zero mass system travelling through zero-mass space, as opposed to the Lorentz factor ( γ ) within the context of special relativity, ^1^ where v is the relative velocity between two reference frames and c the invariant speed of light in vacuum.

In special relativity relativistic effects could be called kinematic in nature, arising from the relative velocity between two space-separated symmetric reference frames. Following this alternative reinterpretation of the Lorentz factor, we should interpret relativistic effects as mechanical in nature, arising from the local relative velocity between a body and its medium. If we take for example time dilation, we could say that physically a clock, simply, is an oscillation of a material system. So when we increase the velocity of a clock relative to the material medium surrounding it, the clock’s oscillation is expected to be retarded. The higher the velocity of a system relative to the medium it is travelling through, the more resistance or inertia it encounters, the more the motions of a clock carried with it will be retarded. At very high velocities the effects on the system will be substantial, even if we consider the medium it’s traveling through to have such a low energy density that we would call it a ‘vacuum’.

In this paper it will be investigated whether we can assume the above and still align with fundamental tests of special relativity.

## Aligning with experiments

### Michelson – Morley

Following special relativity the null result of the Michelson – Morley experiment ^5^ can easily be explained. In a comoving frame the apparatus can be considered at rest, thus the beam travel times are the same.

Following our assumptions the explanation is perhaps as simple. Regardless of their orientation with regard to any space-separated place in the universe, the conditions in both arms the light is travelling through are equal and every part of the apparatus can be considered at rest with regard to its medium. Therefore, the travel time of the split light remains the same and the light remains in phase.

### Kennedy – Thorndike

Kennedy’s and Thorndike’s experiment ^6^ is much the same as Michelson’s and Morley’s. A similar kind of interferometer was used with the difference being that one arm was shorter than the other, and measurements were made throughout the year, to measure the possible effects of the (orbital) velocity of the apparatus relative to a luminiferous ether. This experiment returned a null result as well. It can be explained for by special relativity in much the same manner as Michelson – Morley type experiments.

Again, we also find a simple explanation for experimental results within the framework of our material universe. The lack of fringe shift is easily explained for by considering that the conditions in the apparatus are uniform regardless of the orientation or orbital velocity the apparatus.

Modern variations of Kennedy – Thorndike experiments ^7^ have confirmed the original with much greater precision, but they may be explained for in exactly the same way. We could continue improving the accuracy of Kennedy – Thorndike experiments for years to come, getting the same results with increasing precision and yet they would not allow us to differentiate between the special relativity and our alternative theory.

### Ives – Stilwell

Ives and Stilwell utilized hydrogen canal rays, beams of positive ions, to test if the frequency of light emitted by particles travelling with high velocities would follow classical or relativistic theory.

Their experiments ^8^^,^^9^ did not return a null result. Michelson – Morley and Kennedy – Thorndike experiments could be explained for by any theory that supposes that the motion of Earth through the solar system does not physically influence the measurements made in our laboratories or any theory that supposes it does, but in a manner that prevents us from detecting it. Ives’ and Stillwell’s experimental results are much more solid evidence for special relativity (or Lorentz’ ether theory) because they align with relativistic Doppler shift as opposed to classical Doppler shift. ^8^ As such, they are direct, positive evidence for relativistic time dilation.

Doppler shift, a change in the frequency of waves experienced by the receiver and the source of a signal that are moving in relation to each other can classically be described as such:

$$f_{r}=\frac{\left(v_{w}-v_{r}\right)}{\left(v_{w}+v_{s}\right)}\;f_{s}$$ (1)

Where $f_{r}$ is the observed frequency of the receiver, $v_{w}$ is the speed of propagation of waves in the medium, $v_{r}$ the speed of the receiver relative to the medium, $v_{s}$ is the speed of the source relative to the medium and $f_{s}$ is the frequency observed by the source. The convention used here is that v is negative when source and receiver are approaching each other.

Longitudinal Doppler shift in the context of special relativity can be described ^10^ as such:

$$f_{r}=\frac{1-\beta }{\sqrt{1-\beta^{2}}}\;f_{s}$$ (2)

Where β is the relative velocity between source and receiver in terms of c .

It shows the expression for observed Doppler shift in the context of our theory would have to be different from special relativity. For one, relativistic Doppler shift doesn’t allow for the source and receiver to approach each other faster than c , while our reinterpretation only limits local velocities. Secondly the reference frames of the source and the receiver are not interchangeable like they are in special relativity – they are uniquely dependent on local principle. More specifically, the time dilation a source or receiver experiences is a local phenomenon that relates to their velocity relative to their medium.

**A thought experiment concerning alternative Doppler shift**

Let us imagine a source and a receiver approaching each other through a medium. Both of them have some velocity relative to the medium they are moving through. Both the source and the receiver carry with them a Cesium atomic clock.

They have agreed to record the times at which they send and receive signals and compare these when they meet. To visualize this we could say the source will send a signal, or we could say the waves it emits will crest each 9.192.631.770 periods of its Cesium atomic clock. ^11^ As receiver and source approach or recede from each other the frequency of the signal the receiver measures blue- or redshifts because the distance and time between emission and measurement decrease or increase, following classical Doppler shift. But that is not all: as the source moves through the medium the Cesium atoms’ periods will be retarded compared to when it would be ‘at rest’ relative to the medium. For each second that seems to pass in the frame of the source $1/\sqrt{1-\beta_{s}^{2}}.$ seconds would seem to pass in a frame locally at rest, where $\beta_{s}$ is the velocity of the source relative to the medium in terms of c . Thus the source’s local time, which dictates the frequency by which it sends and records its signals would be observed to be retarded by a factor of $\sqrt{1-\beta_{s}^{2}}$ , and the receiver would observe the frequency by which the source sends out wave crests to decrease by a factor of $\sqrt{1-\beta_{s}^{2}}$ .

Furthermore, the receiver has its own local time dilation dependent on its own velocity relative to the medium. The higher the velocity, the more its own Cesium clock is retarded, and the higher it would observe the frequency by which the signals reach him (by a factor of $1/\sqrt{1-\beta_{r}^{2}}$ ). If receiver and source would meet and compare their logs of signals sent and received, it follows that their results would align with the equation:

$$f_{r}=\frac{1}{\sqrt{1-\beta_{r}^{2}}}\frac{\left(\beta_{w}-\beta_{r}\right)}{\left(\beta_{w}+\beta_{s}\right)}\;\sqrt{1-\beta_{s}^{2}}\;f_{s}=\frac{\sqrt{1-\beta_{s}^{2}}\;}{\sqrt{1-\beta_{r}^{2}}}\frac{\left(\beta_{w}-\beta_{r}\right)}{\left(\beta_{w}+\beta_{s}\right)}\;f_{s}$$ (3)

Where:

•
$f_{r}=$ the frequency observed by the receiver
•
$\beta_{w}=\frac{v}{c}\;$ or the velocity of the wave relative to the medium in terms of c

•
$\beta_{r}=\frac{v}{c}\;$ or the velocity of the receiver relative to the medium in terms of c

•
$\beta_{s}=\frac{v}{c}\;$ or the velocity of the source relative to the medium in terms of c

•
$f_{s}=$ the frequency observed by the source

**Conditional equivalence Lorentz variant alternative and special relativistic Doppler shift**

Equation 3 converges to Equation 1 when considering non-relativistic velocities and it can be shown that it is exactly equivalent to Equation 2 if at least one of the source or the receiver can be considered ‘at rest’ relative to the medium:

Let us consider a source in motion ( $\beta_{s}>0$ ) relative to its medium and a receiver in and at rest relative to the same medium ( $\beta_{r}=0$ ). We assume here that $\beta_{w}\to 1$ so that:

$$f_{r}=\frac{\sqrt{1-\beta_{s}^{2}}}{\sqrt{1-\beta_{r}^{2}}}\frac{\left(\beta_{w}-\beta_{r}\right)}{\left(\beta_{w}+\beta_{s}\right)}\;f_{s}=\frac{\sqrt{1-\beta^{2}}}{1+\beta }f_{s}$$ (4)

Where β is both the local velocity of the source relative to its medium and the global relative velocity between source and receiver. When examining the ratio between the predicted Doppler shifted frequencies of special relativity and the Lorentz variant alternative we find:

$$\frac{f_{r}^{\text{special relativity}}}{f_{r}^{\text{alternative}}}=\frac{\frac{1-\beta }{\sqrt{1-\beta^{2}}}}{\frac{\sqrt{1-\beta^{2}}}{1+\beta }}=\frac{\left(1-\beta \right)\left(1+\beta \right)}{1-\beta^{2}}=\frac{1-\beta +\beta -\beta^{2}}{1-\beta^{2}}=1$$ (5)

And if we consider a receiver in motion and a source at rest, so that $\beta_{r}>0$ and $\beta_{s}=0$ and again assuming that $\beta_{w}\to 1$ , we can see that again Equation 2 and Equation 3 are equal:

$$f_{r}=\frac{\sqrt{1-\beta_{s}^{2}}}{\sqrt{1-\beta_{r}^{2}}}\frac{\left(\beta_{w}-\beta_{r}\right)}{\left(\beta_{w}+\beta_{s}\right)}\;f_{s}=\frac{1-\beta }{\sqrt{1-\beta^{2}}}f_{s}$$ (6)

Where β is both the local velocity of the receiver relative to its medium and the global relative velocity between source and receiver.

**Conditional equivalent Doppler shift in arbitrary direction of motion**

Equation 1– 6 are valid for cases where the velocity of source and receiver can be considered to be aligned with the line between source and receiver, or one could say parallel or antiparallel to the direction of motion of the signal being sent.

*Special relativity*

Within the context of special relativity the observed Doppler shift when considering relative motion in an arbitrary direction can be described as ^1^:

$$f_{r}=\frac{1-\beta \cos\theta_{s}}{\sqrt{1-\beta^{2}}}f_{s}=\gamma \left(1-\beta \cos\theta_{s}\right)f_{s}$$ (7)

Where $\theta_{s}$ is the angle of the line between source - receiver with respect to the velocity of the receiver, as seen from a system of co-ordinates which is at rest relatively to the source.

If we instead describe the relation as seen from a system of co-ordinates which at rest with the receiver the equation is:

$$f_{r}=\frac{\sqrt{1-\beta^{2}}}{1+\beta \cos\theta_{r}}f_{s}=\frac{f_{s}}{\gamma \left(1+\beta \cos\theta_{r}\right)}$$ (8)

Where $\theta_{r}$ is given by the angle between the direction of the line receiver – source and the direction of the velocity of the source.

*Lorentz variant alternative*

Within the context of the Lorentz variant alternative theory discussed in this paper the more generalized form of Equation 3 is described as such:

$$f_{r}=\frac{\sqrt{1-\beta_{s}^{2}}}{\sqrt{1-\beta_{r}^{2}}}\frac{\left(\beta_{w}-\beta_{r}\cos\theta_{r}\right)}{\left(\beta_{w}+\beta_{s}\cos\theta_{s}\right)}$$ (9)

Where $\cos\theta_{r}$ and $\cos\theta_{s}$ are the cosines of the angle of the velocity of the receiver or source relative to the direction of the line source – receiver and receiver – source respectively.

Let $\beta_{s}=0$ and $\beta_{r}>0$ and $\beta_{w}\to 1$ . The alternative theory describes it as such:

$$\;f_{r}=\frac{1-\beta \cos\theta_{r}}{\sqrt{1-\beta^{2}}}f_{s}=\gamma \left(1-\beta \cos\theta_{r}\right)f_{s}$$ (10)

Where β. is both the local velocity of the receiver relative to its medium and the global relative velocity between source and receiver and γ a function of β .

Let $\beta_{r}=0$ and $\beta_{s}>0$ and $\beta_{w}\to 1$ . The alternative theory describes it as such:

$$\;f_{r}=\frac{\sqrt{1-\beta^{2}}}{1+\beta \cos\theta_{s}}f_{s}=\frac{f_{s}}{\gamma \left(1+\beta \cos\theta_{s}\right)}$$ (11)

Where β is both the local velocity of the source relative to its medium and the global relative velocity between source and receiver and γ a function of β .

*Conditional equivalence*

When examining the relation between the angles utilized in special relativity (SR) and the alternative (ALT) one can see that $\theta_{r}^{\mathrm{ALT}}=\theta_{s}^{\mathrm{SR}}$ and $\theta_{s}^{\mathrm{ALT}}=\theta_{r}^{\mathrm{SR}}$ and thus in the case of a source at rest:

$$\;f_{r}^{\mathrm{SR}}=\gamma \left(1-\beta \cos\theta_{s}^{\mathrm{SR}}\right)f_{s}=f_{r}^{\mathrm{ALT}}=\gamma \left(1-\beta \cos\theta_{r}^{\mathrm{ALT}}\right)f_{s}$$ (12)

And in the case of a receiver at rest:

$$\;f_{r}^{\mathrm{SR}}=\frac{f_{s}}{\gamma \left(1+\beta \cos\theta_{r}^{\mathrm{SR}}\right)}=f_{r}^{\mathrm{ALT}}=\frac{f_{s}}{\gamma \left(1+\beta \cos\theta_{s}^{\mathrm{ALT}}\right)}$$ (13)

Due to the relativistic aberration of light the relation between $\cos\theta_{s}^{\mathrm{ALT}}$ and $\cos\theta_{r}^{\mathrm{ALT}}$ and consequently between Equation 12 and 13 is given by the equation ^1^:

$$\cos\theta_{s}^{\mathrm{ALT}}=\frac{\cos\theta_{r}^{\mathrm{ALT}}-\beta }{1-\beta \cos\theta_{r}^{\mathrm{ALT}}}$$ (14)

Interesting to note is that when we set the angle to π/2 we obtain the transverse Doppler effect:

$$\;f_{r}=\frac{f_{s}}{\gamma }=\sqrt{1-\beta^{2}}f_{s}$$ (15)

Where in the context of the alternative theory $\sqrt{1-\beta^{2}}f_{s}$ is a more apt description, because it is the time dilation that the source experiences that is indicative of the change in observed frequency.

**General inequality special relativity and variant Doppler shift**

We can remark that up until now there’s a striking similarity between the predictions made by special relativity and the alternative. But this equivalence ceases to exist when considering (most) cases where both source and receiver can be considered in motion relative to their medium. The difference between the predictions of Equation 9 and those of Equation 7 or 8 becomes more and more pronounced as $\beta ,\;\beta_{r}\;$ and $\beta_{s}$ approach c .

**Aligning with Ives – Stilwell tests**

Ives – Stilwell type experiments have been conducted throughout the years with increasing accuracy ^12^^–^^14^ but as far as the author knows up until now have always involved either a receiver or a source that could be considered at rest relative to the ‘vacuum’ medium surrounding it. They have thus not been able to differentiate between the alternative theory outlined in this paper and special relativity.

## On superluminal velocities

Let us imagine a layered cylinder of matter inside a rest frame medium in the context of the theory outlined in this paper. The outer layer moves with relativistic velocity relative to the ‘rest frame’ medium it is travelling through, and the inner layer is moving with relativistic velocity relative to the outer. Following our assumptions the inner layer would be able to achieve a super-c velocity relative the outer ‘rest frame’ medium, or an observer co-moving with it.

One might argue that this would allow us to observe superluminal velocities, and because we haven’t the outlined theory is disproven. But, already in 1851 Fizeau ^15^ proved that the refraction index of a medium, which is directly related to its energy density, is indicative of the effect of the movement of the medium on the passage of light going through it. Water drags light and other matter more than air does, which drags it more than a ‘vacuum’ does. This shows that in ordinary circumstances we would not observe ‘superluminal’ velocities for even if a ‘vacuum’ medium were to move with relativistic velocity relative to us, it would have a nigh zero effect on light passing through it. And considering the energy required to accelerate a medium of water such that the light passing through it reaches a global super-c velocity, it is clear why we don’t observe this every day on Earth.

Yet we might be observing something just like it in outer space on a regular basis, for example in so called relativistic jets of matter expulsed by active galactic nuclei. These jets of matter have apparent superluminal speeds of up to almost 10 times the speed of light, ^16^ with the innermost parts of the jets attaining the highest speeds while the outermost parts appear slower and are themselves propagating through and interacting with a constant pressure cocoon, either a very hot gas or a magnetized sheath. ^17^ In essence these jets are layered cylinders of matter, with the highest velocities achieved in the inner layers. Precisely the conditions in which you would expect to possibly observe superluminal matter such as light, following our theory.

It follows that Equation 9 is not complete. It still misses a term that would relate to the drag coefficient. We would expect the term to approach 1 for vacuum-like states, and to not be of great concern regarding experiments conducted in ‘vacuum’.

## Testing new physics

To test between the theory outlined in this paper and the special theory of relativity we would need to conduct experiments where both the source and the receiver can be considered to be in motion relative to their supposed medium with some velocity. The experiment would have to be conducted in such a way that we can assume the clocks of the source and receiver to be sufficiently exposed to the medium they’re travelling through.

Curiously, although the predictions of the alternative theory diverge with those of special relativity, the convention currently used to ascertain the accuracy of Ives – Stilwell type experiments cannot be used to differentiate between the two.

Modern Ives – Stilwell tests ^13^^,^^14^ try to experimentally confirm the validity of special relativity’s prediction that:

$$\frac{f_{a}f_{p}}{f_{1}f_{2}}=\frac{1}{\gamma^{2}\left(1-\beta^{2}\right)}=1$$ (16)

Where $f_{a}$ and $f_{p}$ are the frequencies of the lasers propagating antiparallel and parallel to the ion beam and $f_{1}$ and $f_{2}$ are the rest-frame transition frequencies.

The most accurate Ives- Stilwell experiment that has been conducted up until now, by Botermann *et al* in 2014, ^14^ found experiment to align with special relativity’s prediction with an accuracy of $\left|\alpha \right|\leq 2,0\times 10^{-8}$ , where $\alpha =\frac{\varepsilon \left(\beta \right)}{\beta^{2}}$ and $\varepsilon \left(\beta \right)=\sqrt{\frac{f_{a}f_{p}}{f_{1}f_{2}}}-1$

Setting $f_{1}f_{2}$ to unity, we can show that in the case that if at least one of $\beta_{r}=0$ or $\beta_{s}=0$ is true, our outlined theory predicts $\varepsilon \left(\beta \right)=0$ just as special relativity, assuming $\beta_{w}\to c$ :

$$\left(\frac{\sqrt{1-\beta_{s}^{2}}}{1+\beta_{s}\;}\right)\left(\frac{\sqrt{1-\beta_{s}^{2}}}{1-\beta_{s}}\right)=\frac{1-\beta_{s}^{2}}{\left(1+\beta_{s}\right)\left(1-\beta_{s}\right)}=\frac{1-\beta_{s}^{2}}{1-\beta_{s}^{2}}=1$$ (17)

And:

$$\left(\frac{1+\beta_{r}}{\;\sqrt{1-\beta_{r}^{2}}}\right)\left(\frac{1-\beta_{r}}{\sqrt{1-\beta_{r}^{2}}}\right)=\frac{\left(1+\beta_{r}\right)\left(1-\beta_{r}\right)}{1-\beta_{r}^{2}}=\frac{1-\beta_{r}^{2}}{1-\beta_{r}^{2}}=1$$ (18)

As is to be expected considering $f_{a}^{\mathrm{SR}}=f_{a}^{\mathrm{ALT}}$ and $f_{p}^{\mathrm{SR}}=f_{p}^{\mathrm{ALT}}$ .

For the more general case where $\beta_{r}>0$ and $\beta_{s}>0$ it is necessary to employ Equation 3.

Considering that:

$$\begin{array}{c} f_{a}^{\mathrm{ALT}}f_{p}^{\mathrm{ALT}}={\left(\frac{1}{\sqrt{1-\beta_{r}^{2}}}\right)}^{2}\frac{\left(1-\beta_{r}\right)}{\left(1+\beta_{s}\right)}\frac{\left(1+\beta_{r}\right)}{\left(1-\beta_{s}\right)}1-\beta_{s}^{2}\\ ={\left(\frac{1}{\sqrt{1-\beta_{r}^{2}}}\right)}^{2}\frac{1-\beta_{r}^{2}}{1-\beta_{s}^{2}}1-\beta_{s}^{2}\\ =\frac{\left(1-\beta_{s}^{2}\right)\left(1-\beta_{r}^{2}\right)}{\left(1-\beta_{s}^{2}\right)\left(1-\beta_{r}^{2}\right)} \end{array}$$ (19)

It shows that even in the general case the alternative predict *s* $\varepsilon \left(\beta \right)=\sqrt{\frac{f_{a}f_{p}}{f_{1}f_{2}}}-1=0.$

Interestingly, Ives-Stillwell type experiments do provide us with an upper limit of the value of c within the context of the alternative theory. Equation 19 assumes that $\beta_{w}\to c$ and therefore $\beta_{w}=1$ . Following the theory light would never physically be able to reach a velocity equal to c . Since $\beta_{w}\neq c$ , an accurate enough Ives – Stillwell experiment would in theory be able to distinguish between special relativity and the alternative. Looking at the accuracy achieved in 2014, ^14^ $|\alpha |\leq \pm 2\times 10^{-8}$ , and assuming that the velocity of light in our ‘vacuum’ chambers is $v_{\text{light}}=299.792.458\;m/s$ then it follows that $v_{\text{light}}<c\leq \pm 299.792.461\;m/s$.

## Conclusions

This paper shows that by assuming that the universe contains absolutely no free space we can derive an ad hoc reinterpretation and slight alteration of the mathematical framework of the theory of special relativity, that is incompatible with both the postulates of special relativity and with the Galilean principle of relativity, yet can quantitatively account for all fundamental tests of the special theory of relativity.

The outlined model’s predictions diverge with special relativity’s regarding Doppler shift in most situations where both the source and the receiver would be considered in motion relative to their medium, but would exactly align with special relativity’s predictions otherwise.

Of course, the basis of the argument needs to be more thoroughly examined before we follow it to its conclusions. A more thorough investigation is needed to ascertain if there are indeed no tests that have experimentally ruled out the outlined theory, and perhaps a more thorough theoretical examination of the outlined theory is in order. But as of now the idea outlined in this paper seems to show there could be a Lorentz variant theory that is not ruled out by the fundamental tests of special relativity that we have conducted up until now. We could however conduct modified Ives-Stilwell experiments to differentiate between them and to possibly probe for new physics.

## Data Availability

No data are associated with this article.
