# Supplementary material for: A Lorentz variant theory that passes fundamental tests of special relativity and makes diverging, testable but as of yet untested predictions
Source: F1000Res. 2024 Feb 13;12:407. Originally published 2023 Apr 17. [Version 2] doi: 10.12688/f1000research.129133.2 (PMC11318248; doi:10.12688/f1000research.129133.2)
Supplement: Peer review history

# Reviewer report: Reviewer response for version 2

**DOI:** 10.5256/f1000research.162306.r305791

**Published:** 2024-08-08

**Authors:** Jackson Levi Said

**Affiliations:** 1 University of Malta, Msida, Malta

Dear Editor,

 The manuscript attempts to study the potential of an aether-like fluid present in special relativity and more broadly general relativity. The manuscript involves a series of textbook exercises involving three inertial frames and is focused on the influence on the Doppler effect. This additional degree of freedom seems to be poorly motivated both theoretically and experimentally, besides not solving any outstanding problem present in special relativity experiments today. The work needs more theoretical develop, or to be supported by outcomes from data analysis or experiments results.

 For these reasons, I **do not **recommend this manuscript for indexing in its present form. Below, I detail the precise issue to be reconsidered by the authors.

Section 1 - Introduction

 The opening covers some historical aspects of the principal of relativity including how this assumption permeates through Galileo’s work, as well as Newton’s and then in Einstein’s special and general theories of relativity. However, this part of the manuscript then takes a turn and makes unfounded statements such as “no such thing as vacuum” rather than suggesting that these challenges to traditional physics are probes, they are directly stated, making vague whether these are questions to explore or statements to be verified. Moreover, the motivational part has no references to the literature rendering this a rather isolated work. On this point, the terse content in this section makes it hard to understand what framework the author is adopting, as an example, a distinction is made between inertial frames of reference and the background material (air) through which these measurements are being made. My strong suggestion would be to flesh out the motivation for this work, and write a clear research hypothesis that covers the main results of the work.

Section 2 - The basic assumptions

 This short section lays the groundwork for the theoretical development that then takes place in later sections. The core idea is to adopt the aether theory within special relativity where an additional degree of freedom is introduced in the form of the aether dynamics. The motivation or need for this remains an open question. Similarly, a direct measurement also would seem to remain elusive given that the vanilla version of special relativity remains in strong agreement with observational measurements.

Section 3 - Aligning with experiments

 In this section, three classic tests (and some of their variants) are discussed in support of special relativity. More modern tests are not discussed, which is unexpected since their increased precision may provide evidence for or against the proposal in this work. The section then transitions into a discussion of the Doppler effect where a textbook calculation of three frames under this effect is calculated. The next part, similarly, performs a basic limiting exercise to show that as the medium speed decreases, that the equations match. Another standard exercise is then discussed wherein the Doppler effect takes place at an arbitrary angle. This is then generalized for the three frame setting in the next subsection. On this, is this derived from first principles, or simply a substitution of the equations already shown? The section eventually closes with a discussion on conjecture of possible experimental results, why not perform data analysis to assess the strength of any possible such aether-like medium?

Section 4 - Testing new physics

 The hypothetical experiment setting under which this idea could be tested in outlined here. The author finds a best-case scenario in which there would be a marginal deviation from the speed of light but this lacks appropriate uncertainties so it is difficult to assess the statistical significance of the suggestion. The bigger question would be, don’t special relativity experiments already test objects moving relative to a background aether, provided the aether structure that does have such small-scale evolution profiles?

Section 5 - Speculation regarding gravity

 The gravitational part of the manuscript is contained in this section. The author takes the Earth to be stationary with respect to this aether-like medium from the start which seems to negate the underlying proposal of this work. The author then convolutes the Schwarzschild time dilation formula with that in special relativity in Eq. (1). Ultimately, this is an expansion exercise where the leading order acceleration of this possible medium turns out to observe the regular Newtonian acceleration equation, as expected.

Section 6 - Conclusions

 The Conclusion closes with a brief summary of the main observations of the work but is light on quantitative results. This is mainly a discussion of possible future theoretical, data analysis and experimental work.

 Sincerely,

 Referee

Is the work clearly and accurately presented and does it cite the current literature?

Partly

If applicable, is the statistical analysis and its interpretation appropriate?

No

Are all the source data underlying the results available to ensure full reproducibility?

Yes

Is the study design appropriate and is the work technically sound?

No

Are the conclusions drawn adequately supported by the results?

Partly

Are sufficient details of methods and analysis provided to allow replication by others?

Partly

Reviewer Expertise:

The work need more development in terms of the underlying theory, besides at least attempting to perform some data analysis to support the claims made in this work

I confirm that I have read this submission and believe that I have an appropriate level of expertise to state that I do not consider it to be of an acceptable scientific standard, for reasons outlined above.

# Reviewer report: Reviewer response for version 2

**DOI:** 10.5256/f1000research.162306.r299077

**Published:** 2024-08-07

**Authors:** Santanu Raut

**Affiliations:** 1 Mathabhanga College, Cooch Behar, West Bengal, India

This article derives some interesting results assuming that the universe contains absolutely no free space.  Altering the mathematical framework of the theory of special relativity with the Galilean principle of relativity, some tests of special relativity have been conducted over the past century with increasing accuracy and none have showed violations of Lorentz invariance. In the present article, the author examines whether these tests are together sufficient to rule out theories that violate observational symmetry. The author used a variant theory through which relativistic effects such as length contraction and time dilation are purely local consequences. The results are satisfactory for indexing  the article.

Is the work clearly and accurately presented and does it cite the current literature?

Yes

If applicable, is the statistical analysis and its interpretation appropriate?

Not applicable

Are all the source data underlying the results available to ensure full reproducibility?

No source data required

Is the study design appropriate and is the work technically sound?

Yes

Are the conclusions drawn adequately supported by the results?

Yes

Are sufficient details of methods and analysis provided to allow replication by others?

Yes

Reviewer Expertise:

Nonlinear dynamics.

I confirm that I have read this submission and believe that I have an appropriate level of expertise to confirm that it is of an acceptable scientific standard.

# Reviewer report: Reviewer response for version 2

**DOI:** 10.5256/f1000research.162306.r246481

**Published:** 2024-03-04

**Authors:** Roman Szostek

**Affiliations:** 1 Rzeszów University of Technology, Rzeszów, Poland

I believe that the second version of the article

 "A Lorentz variant theory that passes fundamental

 tests of special relativity and makes diverging,

 testable but as of yet untested predictions"

 by Bischoff van Heemskerck D.

 is appropriate.

 The author took my comments into account in a satisfactory manner.

Is the work clearly and accurately presented and does it cite the current literature?

No

If applicable, is the statistical analysis and its interpretation appropriate?

Not applicable

Are all the source data underlying the results available to ensure full reproducibility?

Partly

Is the study design appropriate and is the work technically sound?

Partly

Are the conclusions drawn adequately supported by the results?

Yes

Are sufficient details of methods and analysis provided to allow replication by others?

Yes

Reviewer Expertise:

NA

I confirm that I have read this submission and believe that I have an appropriate level of expertise to confirm that it is of an acceptable scientific standard.

# Reviewer report: Reviewer response for version 1

**DOI:** 10.5256/f1000research.141795.r200395

**Published:** 2023-11-24

**Authors:** Roman Szostek

**Affiliations:** 1 Rzeszów University of Technology, Rzeszów, Poland

The article deals with a very interesting topic, i.e. the mathematical study of a theory that is an alternative to the Special Theory of Relativity and the problem of testing such a theory.

 The work is interesting. In my opinion, the article should be indexed, but after taking into account the following additions and corrections:

**1. **I suggest that the author provide an explicit definition of the principle of relativity. In the 'Introduction' the author discusses this topic, but does not explicitly define the principle of relativity.

**2.** In the chapter " *Michelson-Morley*" the author wrote:

 „ *Regardless of their orientation with regard to any spaceseparated place in the universe, the conditions in both arms the light is travelling through are equal and every part of the apparatus can be considered at rest with regard to its medium*”.

 Why " *can be considered at rest with regard to its medium*"? This statement requires further explanation.

 The same explanation is necessary in the "Kennedy – Thorndike" chapter.

**3.** On page 7 it says "more pronounced as *β*, *β _r_*, and *β _s_* approach *c*".

 This statement requires more detailed comment. After all, *β* = *v*/ *c*. If *β* approach *c*, this means that *v* approach *c* ^2^. Is this really what the author means and what it would mean?

**4.** In the section "On superluminal velocities" the author wrote:

 „ *Following our assumptions the inner layer would be able to achieve a super-c velocity relative the outer ‘rest frame’ medium, or an observer co-moving with it*”.

 It is unclear what the author means by "super-c". The author did not show the formula for summing the speeds in the alternative theory, so it is not obvious what the speed of the inner layer of the cylinder will be. That's why I think this part of the article needs clarification. The formulas for summing the velocities in such alternative theories were derived, for example, in the article [2], which I have provided below.

**5.** The author should provide information about other articles that present research on the same topic.

 There are other studies on relativism with a universal frame of reference, presented in the series of publications [1]-[4]. Numerous models with a universal reference frame have been derived that are consistent with experiments in which the speed of light was measured [1]. The article [2] presents an original method of deriving transformations for kinematics with a universal reference system. The article [3] explains the phenomenon of time dilation in relativistic theories with a universal frame of reference. The article [4] presents a proposal for a mechanical system for testing the Special Theory of Relativity and relativistic theories with a universal frame of reference (Special Theory of Ether).

 [1]  Szostek Karol, Szostek Roman, *The existence of a universal frame of reference, in which it propagates light, is still an unresolved problem of physics*, Jordan Journal of Physics, Vol. 15, № 5, 457-467, 2022, ISSN 1994-7607, https://journals.yu.edu.jo/jjp/JJPIssues/Vol15No5pdf2022/3.html ^1^

 [2]  Szostek Roman, *The original method of deriving transformations for kinematics with a universal reference system*, Jurnal Fizik Malaysia, Vol. 43, Issue 1, 10244-10263, 2022, ISSN 0128-0333, https://www.researchgate.net/publication/368848393 ^2^

 [3]  Szostek Roman, *Explanation of what time in kinematics is and dispelling myths allegedly stemming from the Special Theory of Relativity*, Applied Sciences, Vol. 12(12), 6272, 01-19, 2022, ISSN 2076-3417, https://www.mdpi.com/2076-3417/12/12/6272/htm ^3^

 [4]  Szostek Karol, Szostek Roman, *The concept of a mechanical system for measuring the one-way speed of light, Technical Transactions*, Tom 120, No. 2023/003, e2023003, 1-9, 2023, ISSN 2353-737X, https://sciendo.com/pl/article/10.37705/TechTrans/e2023003 ^4^

Is the work clearly and accurately presented and does it cite the current literature?

No

If applicable, is the statistical analysis and its interpretation appropriate?

Not applicable

Are all the source data underlying the results available to ensure full reproducibility?

Partly

Is the study design appropriate and is the work technically sound?

Partly

Are the conclusions drawn adequately supported by the results?

Yes

Are sufficient details of methods and analysis provided to allow replication by others?

Yes

Reviewer Expertise:

Special Theory of Relativity, Special Theory of Ether, Mechanics, applications of mathematics, cosmology

I confirm that I have read this submission and believe that I have an appropriate level of expertise to confirm that it is of an acceptable scientific standard, however I have significant reservations, as outlined above.

## References

[1] : The Existence of a Universal Frame of Reference, in Which it Propagates Light, is Still an Unresolved Problem of Physics. *Jordan Journal of Physics* .2022; Reference source

[2] : The original method of deriving transformations for kinematics with a universal reference system. *Jurnal Fizik Malaysia* .2022; Reference source

[3] : Explanation of What Time in Kinematics Is and Dispelling Myths Allegedly Stemming from the Special Theory of Relativity. *Applied Sciences* .2022;12(12) : 10.3390/app12126272 10.3390/app12126272

[4] : The concept of a mechanical system for measuring the one-way speed of light. *sciendo* .2023; Reference source

# Response

**Published:** 2024-02-05

**Authors:** Daniël Bischoff van Heemskerck

**Affiliations:** Leiden University, The Netherlands

First of all I'd like to thank you for spending your valuable time to review my work, it is very much appreciated!

 1. I have included a more explicit definition of the principle of relativity in the introduction.

 2. You are completely right in that the assumptions regarding the dynamics between the medium and the apparatus or lack thereof should be more thoroughly explained. I have provided a more thorough explanation of the physical picture of the 'vacuum medium' that is assumed in my paper in the introduction, and explained why it is as natural to assume the medium is at rest with the apparatus as to assume it isn't in the Michelson Morley section. For the sake of completeness, I also included a section near the end of the paper which considers a scenario where the apparatus and the medium are not at rest, but their relative motion uniform regardless of Earth's orbit by considering gravity.

 3. Perhaps as you suspected: A mistake on my part, I meant to say as *β *approaches 1, or *v* approaches *c*. Thanks for catching this mistake.

 4. I have tried to explain more thoroughly what I meant by super-c velocities and have included some studies in the field to highlight the potential need for a theory that allows for global velocities that exceed the speed of light. I'm not sure if the transformations you've provided, their merit notwithstanding, could be extended without additional considerations to these superluminal jets, whose dynamics might not be well suited to be treated purely kinematically, although it might be an exciting topic for future work. For the sake of simplicity I would just want to state here that if we're relieved of SR's second postulate, we could in theory observe global velocities greater than the speed of light, but still we would expect there to be physical, local limits. Therefore these layered cylinders are places where we could expect to possibly observe these high velocities. Although of course, as you posit, even from the perspective of SR one could argue that c should not necessarily be treated as an universal, global limit.

 5. Thank you for introducing me to your work! I have included a reference to your work on a physical explanation for time dilation, which I think is a very fundamental approach that aligns with the investigation in my paper, and should indeed be referenced. I have made a preliminary inquiry into formulating time dilation by treating a clock as an underdamped harmonic oscillator, whose damped frequency of oscillation is given by *ω* *_1* *=* *ω* *_0* *sqrt(1-ζ^2) *and the damping ratio *ζ∝ v/c. *When the relative velocity between the medium and the clock reaches c the oscillator becomes critically damped, and no longer functions as a clock, much like the light clock considered in your paper. Perhaps sometime we could discuss further.

 Thank you again for your time and consideration,

 DMF Bischoff van Heemskerck
